# Artificial intelligence assistance for fetal development: evaluation of an automated software for biometry measurements in the mid-trimester

**DOI:** 10.1186/s12884-024-06336-y

**Published:** 2024-02-23

**Authors:** Xuesong Han, Junxuan Yu, Xin Yang, Chaoyu Chen, Han Zhou, Chuangxin Qiu, Yan Cao, Tianjing Zhang, Meiran Peng, Guiyao Zhu, Dong Ni, Yuanji Zhang, Nana Liu

**Affiliations:** 1 Department of Ultrasonography, Shenzhen University General Hospital, Shenzhen, Guangdong China; 2 National-Regional Key Technology Engineering Laboratory for Medical Ultrasound, School of Biomedical Engineering, Shenzhen University Medical School, Shenzhen University, Shenzhen, Guangdong China; 3 Medical Ultrasound Image Computing (MUSIC) Lab, Shenzhen University, Shenzhen, Guangdong China; 4 Marshall Laboratory of Biomedical Engineering, Shenzhen University, Shenzhen, Guangdong China; 5 Shenzhen RayShape Medical Technology Co., Ltd, Shenzhen, Guangdong China; 6 NVIDIA, Shenzhen, China

**Keywords:** Biometry measurement, Artificial intelligence, Fetal growth and development

## Abstract

**Background:**

This study presents CUPID, an advanced automated measurement software based on Artificial Intelligence (AI), designed to evaluate nine fetal biometric parameters in the mid-trimester. Our primary objective was to assess and compare the CUPID performance of experienced senior and junior radiologists.

**Materials and methods:**

This prospective cross-sectional study was conducted at Shenzhen University General Hospital between September 2022 and June 2023, and focused on mid-trimester fetuses. All ultrasound images of the six standard planes, that enabled the evaluation of nine biometric measurements, were included to compare the performance of CUPID through subjective and objective assessments.

**Results:**

There were 642 fetuses with a mean (±SD) age of 22 ± 2.82 weeks at enrollment. In the subjective quality assessment, out of 642 images representing nine biometric measurements, 617-635 images (90.65-96.11%) of CUPID caliper placements were determined to be accurately placed and did not require any adjustments. Whereas, for the junior category, 447-691 images (69.63-92.06%) were determined to be accurately placed and did not require any adjustments. In the objective measurement indicators, across all nine biometric parameters and estimated fetal weight (EFW), the intra-class correlation coefficients (ICC) (0.843-0.990) and Pearson correlation coefficients (PCC) (0.765-0.978) between the senior radiologist and CUPID reflected good reliability compared with the ICC (0.306-0.937) and PCC (0.566-0.947) between the senior and junior radiologists. Additionally, the mean absolute error (MAE), percentage error (PE), and average error in days of gestation were lower between the senior and CUPID compared to the difference between the senior and junior radiologists. The specific differences are as follows: MAE (0.36-2.53 mm, 14.67 g) compared to (0.64- 8.13 mm, 38.05 g), PE (0.94-9.38%) compared to (1.58-16.04%), and average error in days (3.99-7.92 days) compared to (4.35-11.06 days). In the time-consuming task, CUPID only takes 0.05-0.07 s to measure nine biometric parameters, while senior and junior radiologists require 4.79-11.68 s and 4.95-13.44 s, respectively.

**Conclusions:**

CUPID has proven to be highly accurate and efficient software for automatically measuring fetal biometry, gestational age, and fetal weight, providing a precise and fast tool for assessing fetal growth and development.

## Background

Accurate biometric measurements conducted on ultrasound images enable the evaluation of fetal normality, including the estimation of fetal size, gestational age (GA), and estimated fetal weight (EFW) [1–4]. This differentiation is important for distinguishing between fetal size at a given timepoint and fetal growth [5, 6]. Ultrasound measurements can also facilitate the identification of abnormalities. They can detect developmental abnormalities of individual organs, such as the central nervous system (CNS) [7, 8], skeletal and limb systems [9], uneven development, as well as overall developmental abnormalities like fetal growth restriction (FGR), small for gestational age (SGA), and large for gestational age (LGA) [6, 10, 11]. Comprehensive measurements can help in making informed decisions regarding the fetus, including potential interventions, intrauterine therapy, or even the option of pregnancy termination [12, 13]. Since the accuracy of biometric measurements depends heavily on the operator’s expertise [14], it results in poor consistency in biometric measurements, and potential diagnostic errors [15]. Moreover, operators can cause repetitive stress injuries through multiple keystrokes and are time-consuming [16], especially during refined mid-trimester measurements to assess fetal growth and development [17–20].

We have developed an advanced automatic measurement software named CUPID (RayShape, China), a mature commercial product designed to eliminate inter-observer variability, reduce repetitive stress injuries, and save time in fetal biometric measurements. To improve its computational performance, we deployed CUPID on the Nvidia Clara AGX development kit with RTX 6000 using TensorRT. The CUPID system could recognize six standard planes required by the guidelines including the transthalamic, transventricular, transcerebellar, abdominal circumference, femur, and humerus planes for automatically measuring nine biometric parameters containing biparietal diameter (BPD), head circumference (HC), occipitofrontal diameter (OFD), transverse cerebellar diameter (TCD), posterior cranial fossa pool width (PCFW), lateral ventricles width (LVW), abdominal circumference (AC), femoral length (FL) and humeral length (HL) [21, 22]. In addition, CUPID incorporates the capability to calculate GA and EFW using the formulas [10, 23–27].

The main objective of this pilot study was to assess CUPID’s performance and efficiency in measuring the fetus’s nine biometric parameters compared to two radiologists with different levels of experience.

## Materials and methods

### Data collection

**Fig. 1:**
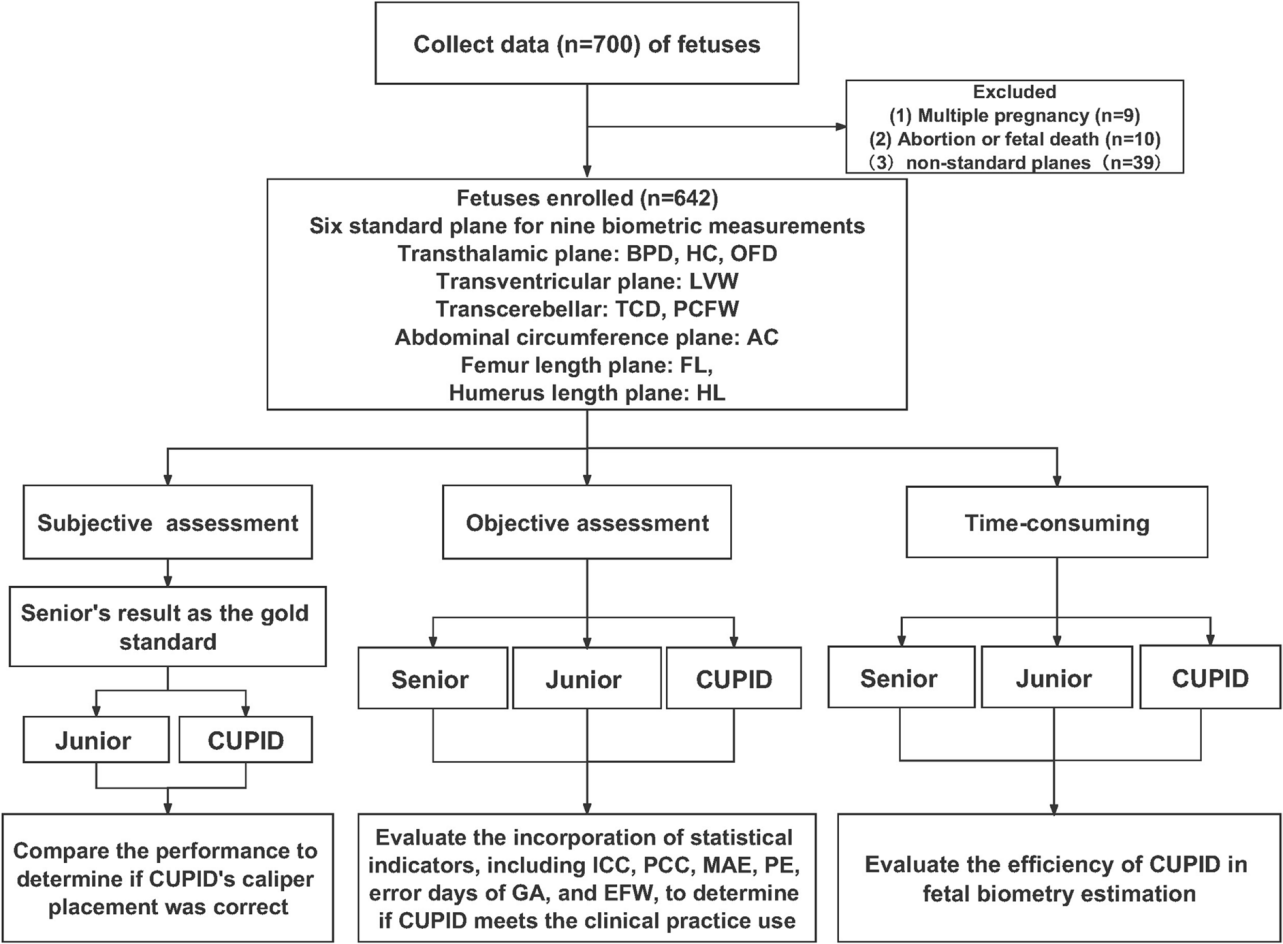
Flowchart summarizing the study design

A prospective cross-sectional study was conducted at Shenzhen University General Hospital between September 2022 and June 2023 in which 700 pregnant women in their mid-trimester were enrolled, as shown in Fig. 1. Only women with healthy singleton pregnancies and a certain fetal crown-rump length were included in the study. All examinations were performed by two senior radiologists with over 10 years of experience in obstetrics, using GE Voluson E8/E10 ultrasound machines (GE Healthcare, Zipf, Austria) equipped with C1-6 probes. The collected patient measurement data comprised six standard planes: transcranial, transthalamic, transcerebellar, abdominal circumference, femur, and humerus. We implemented quality control on the collected images, for which two expert radiologists with over 20 years of experience not involved in data collection evaluated the six standard planes following the ISUOG guidelines. Specific evaluation criteria included complete anatomy, appropriate size, and high image quality to ensure optimal imaging plane acquisition [2]. The evaluation results included both standard and non-standard planes, and only images simultaneously rated as standard planes by both experts were further included in the study. After quality control process, a total of 642 cases were enrolled. The Research Ethics Committee of Shenzhen University General Hospital approved the study, and informed consent was obtained from all women.

### Study design

We designed our study based on a validation dataset obtained after a rigorous review process. This study involved conducting independent and comprehensive data collection within the setting of Shenzhen University General Hospital, without modifying the algorithm. Biometric measurements were taken twice at two-week intervals by both the manual and automatic groups, following the standards defined by ISUOG Practice Guidelines [2, 6, 21]. The manual group comprised two radiologists: an experienced senior obstetric radiologist (N. Liu, Senior) with over 10 years of expertise, and a junior radiologist (X. Han, Junior) with 3 years of experience in obstetrics. All manual group measurements have been annotated manually by Senior and Junior using the Pair [28] annotation software package. The automatic group inputs the corresponding images into the CUPID software, which automatically obtains the measurements and estimates the GA and fetal weight. The performances of the manual and automatic groups were studied concerning the following measurements: BPD, OFD, HC, LVW, TCD, PCFW, AC, FL, HL, GA and EFW. All examiners were blinded to the measurements obtained during ultrasound examination. The measurements of the Senior (N. Liu) were used as the gold standard to compare the performance of the Junior (X. Han) and CUPID.

**Fig. 2:**
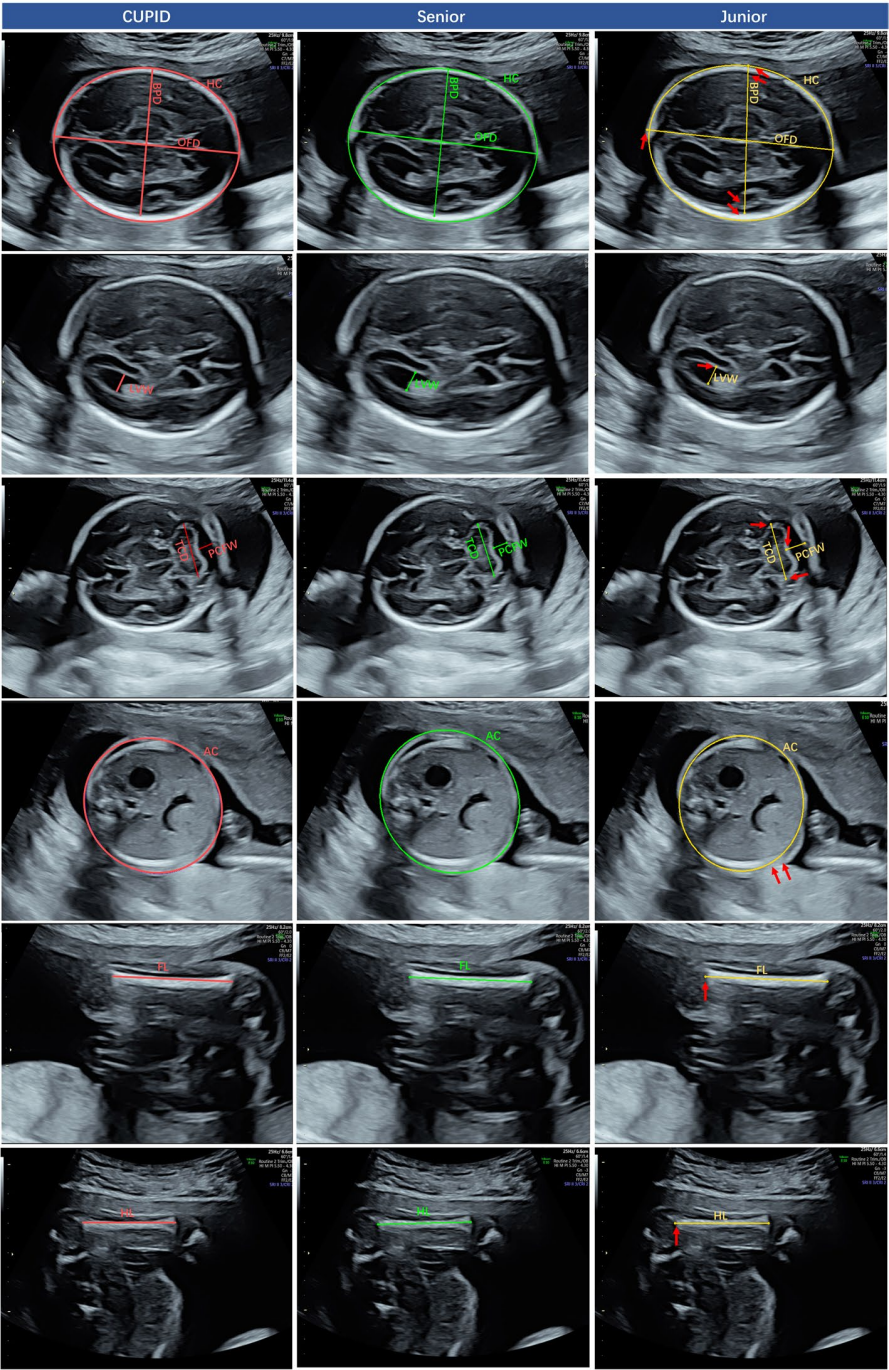
Examples of measurement results obtained by Senior, Junior and CUPID for the nine key fetal biometric parameters. BPD, biparietal diameter; HC, head circumference; OFD, occipitofrontal diameter; TCD, transverse cerebellar diameter; PCFW, posterior cranial fossa pool width; LVW, lateral ventricles width; FL, femoral length; HL, humeral length; AC, abdominal circumference (The red arrow indicates the location of the measurement error)

**Fig. 3:**
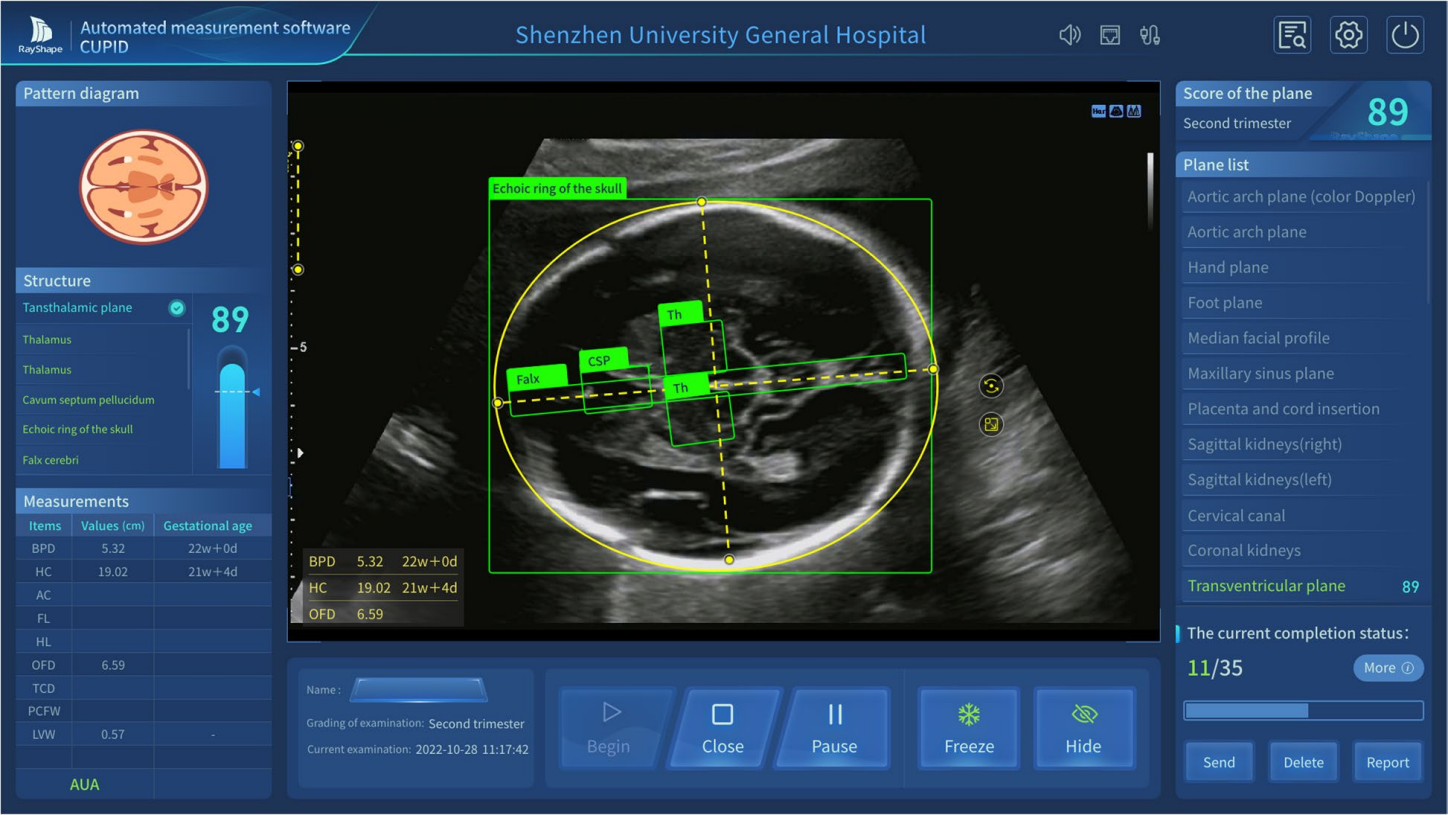
CUPID’s product interface. BPD, biparietal diameter; HC, head circumference; OFD, occipitofrontal diameter; Th, thalamus; CSP, cavum septum pellucidum; Falx, falx cerebri

An annotation process was conducted in three distinct phases. In phase 1, a subjective clinical assessment was performed once on each image by Senior to determine whether the caliper placement of the CUPID and Junior was correct. Caliper position was classified as either a good fit or an adjustment required [11]. In phase 2, we performed objective assessments to compare the consistency and relative error between radiologists with different seniority levels and CUPID for the biometric measurements. These measurements included BPD, OFD, HC, LVW, TCD, PCFW, AC, FL, and HL. This evaluation aimed to determine whether CUPID met clinical practice standards. Examples of the manual and automatic measurement results for the nine biometric measurements and CUPID’s product interface are shown in Figs. 2 and 3. In addition, the measured values of BPD, HC, TCD, AC, and FL were used to determine the gestational age and estimated fetal weight based on the Hadlock formula [23]. In phase 3, the time-consuming to measure each parameter was recorded for manual and automatic groups, enabling a comprehensive evaluation of the efficiency of CUPID in fetal biometric estimation.

### The development of commercial CUPID software

To develop CUPID into a mature commercial software, we initially conducted tasks such as model training and performance evaluation. The dataset used for training CUPID’s artificial intelligence algorithm was independently collected and established at different stages of software development. The algorithm employed by CUPID is based on a traditional convolutional neural network architecture. Dataset used to train CUPID encompasses 5000 cases, divided into training, validation, and test sets in a 7:1:2 ratio. After rigorous preliminary clinical trials conducted across multiple centers, we found that CUPID’s performance in nine key measurement items is highly consistent with manual measurements by experts, with a consistency coefficient exceeding 0.9. To enhance the performance of CUPID, we opted to deploy the CUPID software on the Nvidia Clara AGX developer kit^1^ with RTX 6000. The kit was purpose-built for medical instruments that require advanced computation to support various real-time workloads that come with a fully tested operating system and drivers. To improve the performance further, Nvidia TensorRT techniques are also adopted in product settings. These techniques help successfully achieve twice the acceleration compared to the ONNXRuntime architecture [29].

### Statistical analysis

This study utilized intra- and inter-class correlation coefficients (ICC), Pearson correlation coefficient (PCC), and Bland-Altman (BA) plots to assess repeatability and reproducibility. The mean absolute error (MAE) and percentage error (PE) were also used to understand the variability associated with individual measurements. A paired Welch’s two-sample test was used to analyze the statistical significance between the groups, and a significance level of < 5% was considered statistically significant. Statistical analyses were performed using SPSS version 26.0.

## Results

### Characteristics of the study

A total of 642 fetuses were recruited after providing consent for the study. The mean gestational age of the fetuses was 22 weeks ±2.82 (SD) (range: 18-24 weeks gestation). Table 1 lists the factors related to pregnant women and fetus characteristics. Maternal age, median cervical canal length, history of cesarean section, previous open abdominal surgery, and prior laparoscopic abdominal surgery have also been reported. Additionally, the fetus information comprises gestational week, placental position, and the deepest vertical pocket of amniotic fluid (DVP).

**Table 1:** Clinical characteristics of 642 patients undergoing routine prenatal screening in mid-trimester (18-24 weeks of gestation)

**Pregnant women**	n = 642
Mean Maternal age (years)	30(±3)
Cervical canal length (mm)	51(±12)
History of cesarean section	12
Previous open abdominal surgery	10
Prior laparoscopic abdominal surgery	13
**Fetal**	n = 642
**Gestational week**	
18 weeks	5
19 weeks	13
20 weeks	39
21 weeks	207
22 weeks	265
23 weeks	95
24 weeks	18
**Placental position**	
Posterior	251
Anterior	258
Fundal or lateral	133
**Amniotic fluid anteroposterior diameter (mm)**	47 ± 5

### The intra-observer reproducibility

Intra-observer reproducibility was assessed for measuring 5778 biometric variables derived from 642 fetuses. As shown in Table 2, the ICC for Senior, Junior and CUPID were 0.974-0.999, 0.749-0.934 and 1.0 respectively. High ICC values demonstrated excellent agreement and consistency between repeated measurements, indicating strong intra-observer reproducibility.

**Table 2:** Intra-observer reproducibility of nine key fetal biometric parameters by a Senior, Junior, and CUPID

Parameters	Senior	Junior	CUPID
	ICCs	ICCs	ICCs
BPD	0.997	0.889	1.000
HC	0.974	0.934	1.000
OFD	0.967	0.892	1.000
LVW	0.975	0.769	1.000
TCD	0.999	0.749	1.000
PCFW	0.999	0.769	1.000
AC	0.994	0.789	1.000
FL	0.999	0.898	1.000
HL	0.999	0.908	1.000

### Subjective clinical assessment

In the subjective quality assessment of 642 fetuses representing nine biometric measurements, there were 617-635 images (90.65-96.11%) of CUPID caliper placements deemed a good fit and did not require any adjustment. Similarly, 447-691 images (69.63-92.06%) were determined as a good fit and did not require any adjustment for the Junior (Table 3).

**Table 3:** Subjective assessment of the clinical acceptability of caliper placement by CUPID and junior for measuring nine biometric parameters (*n* = 642), n denotes the number of participants

Parameters	CUPID		Junior	
	Good fit	Adjustment	Good fit	Adjustment
BPD	617 (96.11%)	25 (3.89%)	585 (91.12%)	57(8.88%)
HC	617 (96.11%)	25 (3.89%)	583 (90.81%)	59 (9.19%)
OFD	617 (96.11%)	25 (3.89%)	586 (91.28%)	56 (8.72%)
LVW	617 (96.11%)	25 (3.89%)	447 (69.63%)	195 (30.37%)
TCD	585 (91.12%)	57 (8.88%)	471 (73.36%)	171 (26.64%)
PCFW	582 (90.65%)	60 (9.35%)	460 (71.65%)	182 (28.35%)
AC	606 (94.39%)	36 (5.61%)	562 (87.54%)	80 (12.46%)
FL	615 (95.79%)	27 (4.21%)	563 (87.69%)	79 (12.31%)
HL	613 (95.79%)	29 (4.52%)	591 (92.06%)	51 (7.94%)

### Objective assessment

**Fig. 4:**
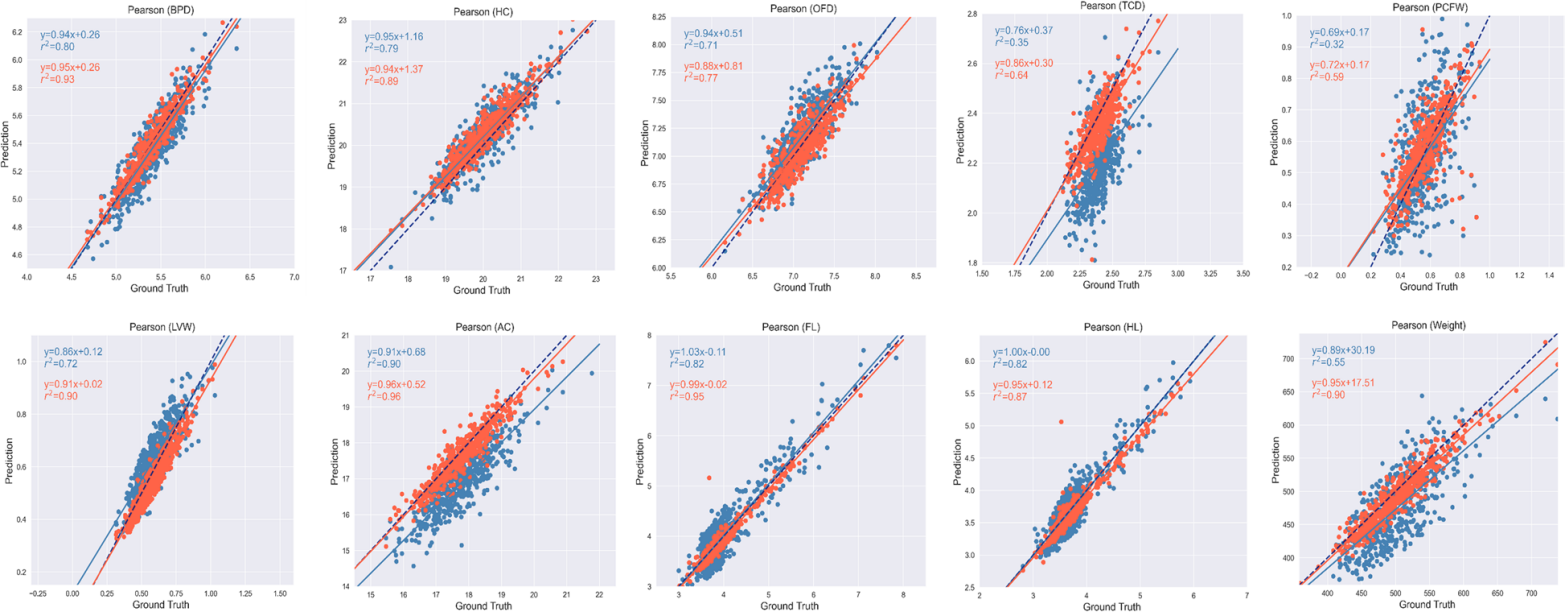
The Pearson correlation coefficient plot shows the agreement between Senior and CUPID, as well as between Senior and Junior, regarding the measurement of nine key fetal biometric parameters and EFW (blue dashed line represents Senior, red line represents CUPID, and blue solid line represents Junior)

**Fig. 5:**
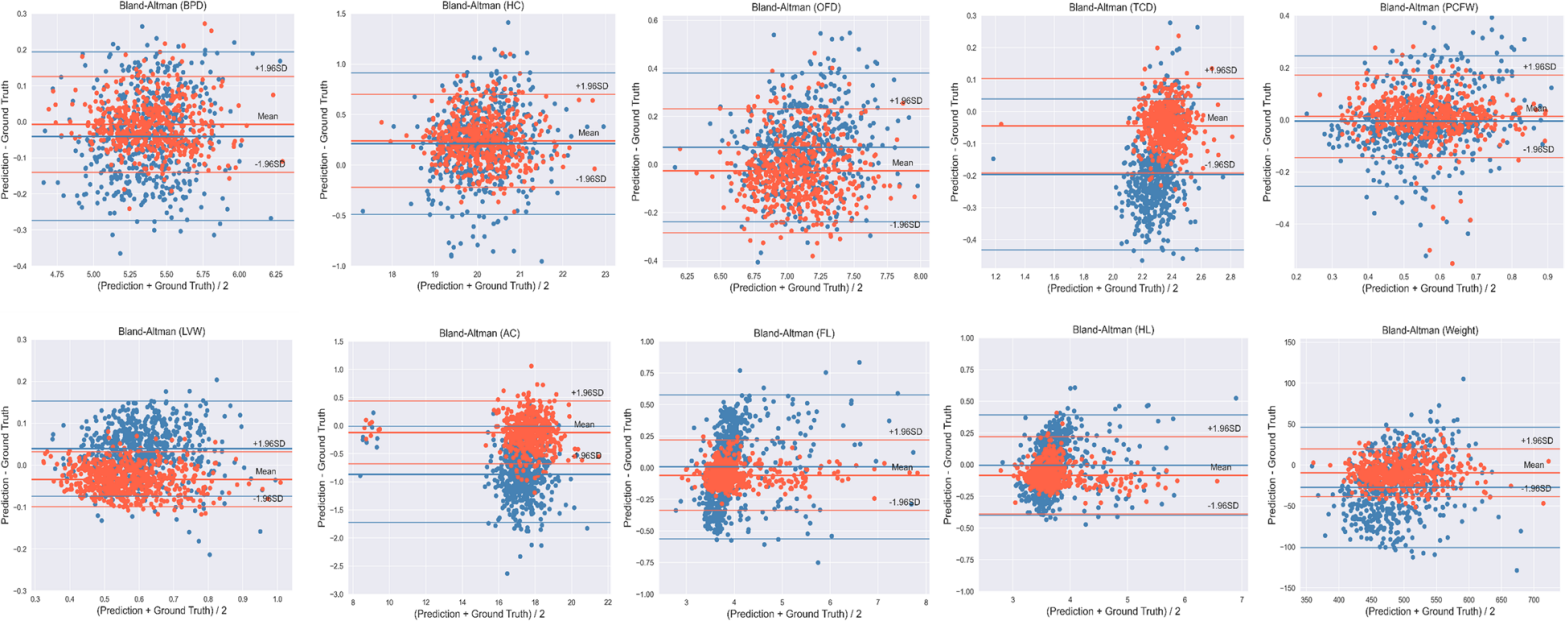
The Bland-Altman plot shows the agreement between Senior and CUPID, as well as between Senior and Junior, regarding the measurement of nine key fetal biometric parameters and EFW (red dots: senior and CUPID, blue dots: Senior and Junior)

**Fig. 6:**
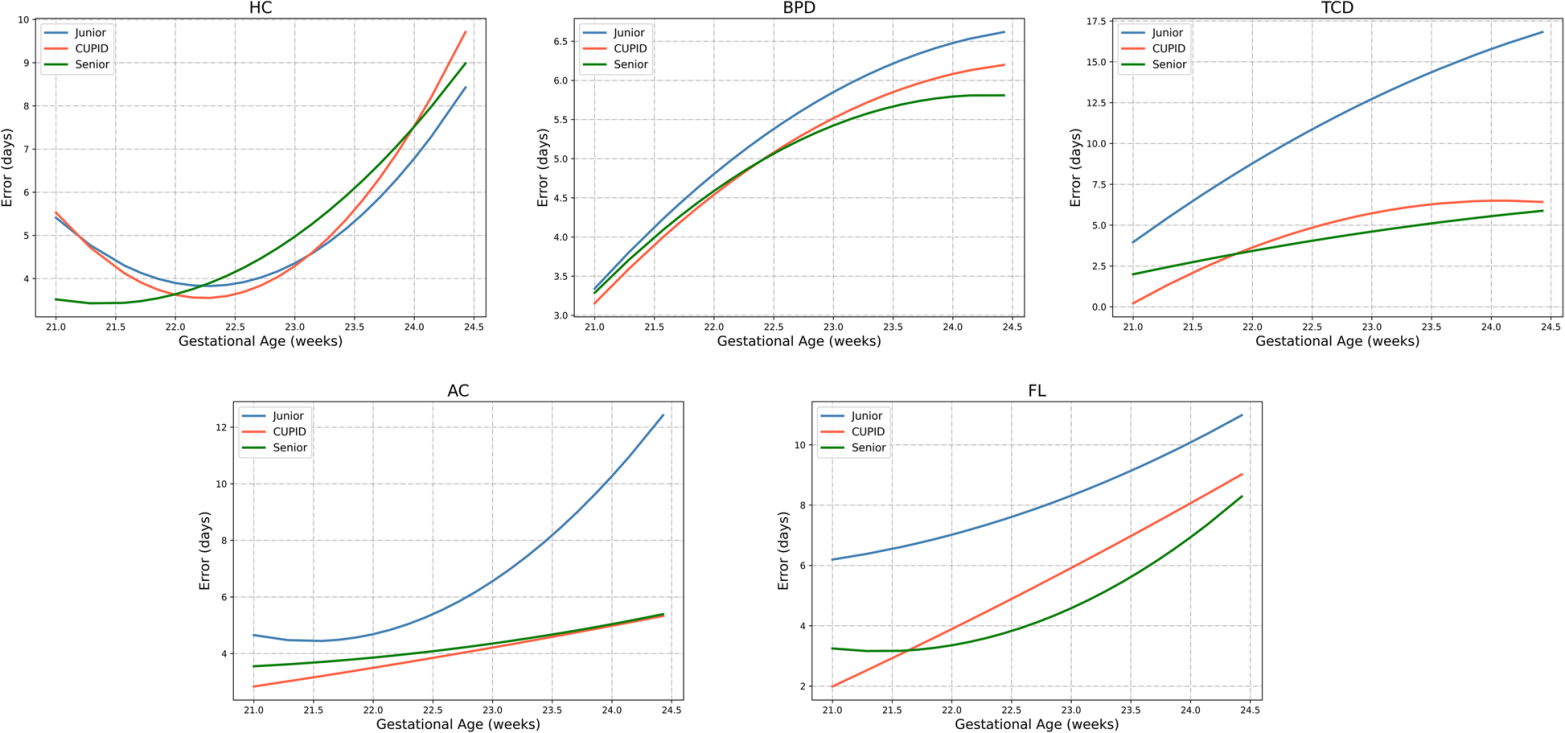
Trend curve of estimated gestational age error in days measured by CUPID, Senior, and Junior

The objective measurement indicators assessed the inter-observer reproducibility of measurements for 5778 biometric variables derived from 642 fetuses. Table 4 presents the results of the Senior, Junior and CUPID on the nine fetal biometric measurements and EFW, and we can find that for most of the measured results, CUPID is closer to Senior compared to Junior. The ICC, PCC, MAE, and PE values are presented in Table 5. The ICC between the Senior and CUPID (0.843-0.990) reflected better reliability than between the Senior and Junior (0.306-0.937). Meanwhile, PCC showed the same results as the ICC, demonstrating that the CUPID results have a higher linear correlation with Senior. In addition, the results for MAE and PE in Table 5 again demonstrate the reliability of CUPID. Figure 4 illustrates the correlation distribution map of the nine biometric measurements and EFW. The agreement distribution of all measurements was shown in Fig. 5. From Fig. 5, we can clearly find that CUPID and Senior are more consistent, especially for the measurement items TCD, LVW, AC, FL, HL and EFW. The comparisons of error days in the true gestational age for Senior, Junior and CUPID were in Table 6, and Fig. 6 illustrates the error curve for gestational age estimation. CUPID also showed superior performance in estimating gestational age compared to Junior. In conclusion, these results clearly demonstrate a high consistency and correlation between the measurements obtained through CUPID and Senior.

**Table 4:** Measurement results of nine key fetal biometric parameters and EFW present with the mean ± SD format. The nine key fetal biometric parameters in mm, and EFW in g

Parameters	Senior	Junior	CUPID
BPD	53.93 ± 2.53	53.64 ± 2.56	53.84 ± 2.47
HC	199.92 ± 7.14	202.03 ± 7.67	202.33 ± 7.12
OFD	70.92 ± 2.64	71.64 ± 2.93	70.66 ± 2.66
LVW	5.90 ± 1.50	6.30 ± 1.07	5.47 ± 1.06
TCD	23.85 ± 1.14	21.89 ± 1.46	23.41 ± 1.22
PCFW	5.59 ± 1.21	5.55 ± 1.48	5.73 ± 1.14
AC	176.48 ± 13.61	167.81 ± 13.10	175.28 ± 13.41
FL	39.69 ± 5.96	39.76 ± 6.78	39.10 ± 6.07
HL	37.51 ± 4.28	37.48 ± 4.74	36.68 ± 4.33
EFW	491.45 ± 46.80	473.65 ± 55.57	500.98 ± 46.98

**Table 5:** Quantitative evaluation of nine key fetal biometric parameters and EFW. (ICC: inter-class correlation coefficients, PCC: Pearson correlation coefficient, MAE: mean absolute error, PE: percentage error)

Parameters	Senior and CUPID				Senior and Junior			
	ICC	PCC	MAE (mm)	PE (%)	ICC	PCC	MAE (mm)	PE (%)
BPD	0.982	0.975	0.50	0.94	0.937	0.931	0.96	1.79
HC	0.895	0.972	2.53	1.28	0.849	0.942	3.13	1.58
OFD	0.946	0.931	1.03	1.46	0.928	0.915	1.26	1.78
LVW	0.904	0.950	0.36	6.14	0.797	0.851	0.64	11.59
TCD	0.843	0.799	0.55	2.29	0.306	0.595	1.96	8.22
PCFW	0.877	0.765	0.47	9.38	0.661	0.566	0.81	16.04
AC	0.982	0.978	2.22	1.25	0.797	0.947	8.13	9.88
FL	0.990	0.972	0.81	1.99	0.901	0.904	2.42	13.23
HL	0.970	0.935	0.96	2.57	0.916	0.904	1.64	4.37
EFW	0.943	0.894	14.67(g)	2.92	0.671	0.741	38.05(g)	7.56

**Table 6:** Comparison of error days in true gestational age for Senior, Junior and CUPID

Parameters	Average error days of gestation		
	Senior	Junior	CUPID
HC	4.15	4.35	3.99
BPD	5.56	5.90	5.59
TCD	4.43	11.06	5.26
FL	4.44	7.92	5.67
AC	4.73	5.86	4.45

### Time-consuming

Table 7 summarizes the time-consuming process of measuring each parameter. It reveals that CUPID demonstrated a faster measurement time for BPD, OFD, HC, LVW, TCD, PCFW, AC, FL, and HL with a range of 0.05-0.07 s. This faster measurement time is advantageous over Senior (4.79-11.68 s) and advantage over Junior (4.95-13.44 s).

**Table 7:** A summary of the time-consuming to measure each parameter by Senior, Junior, and CUPID in seconds

Parameters	Time-consuming (s)			
	Senior	Junior	CUPID	***P***
BPD	5.10	6.03	0.06	<0.01
HC	10.66	12.34	0.06	<0.01
OFD	5.04	6.51	0.06	<0.01
LVW	7.89	7.90	0.06	<0.01
TCD	4.79	4.95	0.05	<0.01
PCFW	6.77	6.79	0.06	<0.01
AC	11.68	13.44	0.06	<0.01
FL	9.38	9.51	0.06	<0.01
HL	9.53	9.99	0.07	<0.01

## Discussion

Fetal growth and development are essential aspects of antenatal care. Fetal ultrasound plays an important role in assessing these conditions through multiple biometric measurements, that rely on the expertise of the operator [6]. We successfully developed a novel artificial intelligence assistance software, CUPID, to automatically measure nine crucial fetal biometric variables obtained in the mid-trimester. In this comprehensive comparative study, we evaluated the placement of CUPID and the accuracy of its measurements to determine its precision in fetal biometry of standard plane and its predictive ability for GA and EFW. The study analyzed images that had already undergone quality control. It included radiologists of different seniority levels, and CUPID consistently outperformed Junior while approaching Senior’s performance levels across all biometric measurements. CUPID system stands out in the terms of efficiency as it can measure all nine biometric in less than 1 second. It is a highly efficient and advantageous option for saving measurement time.

Inconsistencies in doctors’ skills can result in measurement and diagnostic errors, emphasizing the significance of quality control in fetal biometric data. Quality control measures in this context involve assessing intra- and interobserver reproducibility through caliper placement on stored images by the same and different operators. CUPID demonstrates good intra-observer reproducibility consistently producing the same results when performing multiple measurement operations on the same image. Conversely, doctors’ operations are closely related to seniority, and junior doctors show poor intra-observer consistency. CUPID performed significantly better than the junior doctors in the inter-observer reproducibility experiment. The CUPID was considered a good fit for more than 90% of the image caliper placement positions, as evaluated by Senior. The junior’s limited experience affects their performance, as they struggle with measurements of specific organs and soft tissues, such as LVW, TCD, PCFW, and AC. Nevertheless, Junior can adequately perform measurements with clear boundaries, such as HC, BPD, OFD, FL, and HL.

Automated measurements conducted in the mid-trimester offer a valuable means of enhancing the dependability of various assessments [11, 30]. We found that CUPID maintained a high degree of consistency with the Senior in measurements, and when compared with true gestational age, the results of HC and AC were better than those of Senior’s. The analysis found that this may be because when doctors perform HC or AC measurements, they employ ellipse fitting [12]. In contrast, CUPID is fitted through the complete boundaries of HC or AC, which is closer to actual development. Similarly, we evaluated CUPID’s measurement accuracy for subtle intracranial structures, which are the indicators that need to be assessed during the mid-trimester. CUPID’s performance was excellent because it could accurately identify the lateral ventricles and cerebellum, providing diagnostic assistance for potential CNS conditions. However, its performance in measuring PCFW was slightly inferior to other indicators. The analysis revealed that PCFW has a high degree of structural variability, which making it more challenging for AI to learn. However, Junior’s performance in these subtle structures was extremely poor because Junior lacked experience correctly identifying and measuring them. Junior consistently measured the cerebellum as smaller and the lateral ventricles as larger, which could potentially lead to false positives and unnecessary examinations during clinical diagnosis. Therefore, by comparing the consistency of different years of experience and CUPID in all nine biometric measurements, we found that CUPID’s performance is closer to Senior, and better in some measurement items. All CUPID measurements showed an error of less than 6 days compared to the true gestational age. According to the literature, a predictive error of ±10 days for gestational age in the mid-trimester is acceptable in the most clinical settings [31]. It takes approximately 0.5 seconds to perform all nine fetal biometric measurements using CUPID. Therefore, CUPID is reliable and reproducible automated software for clinical applications. The use of CUPID can reduce work-related musculoskeletal disorders resulting from repetitive movements.

It is important to acknowledge the limitations of this study despite these compelling findings. Primarily, our investigation was confined to fetuses exhibiting normal intracranial anatomy, which may constrain the broad applicability of our findings to a diverse population. Second, since this study was conducted in a single-center setting, it is necessary to conduct further multicentric validation to ascertain the universal applicability of CUPID’s AI measurement across multiple ethnicities and geographical demographics. Finally, this study did not perform a direct comparison between CUPID’s and radiologists’ measurements on images that were not subjected to quality control; this might have led to missing some of the real clinical situations in which the two performances were compared.

In conclusion, the CUPID software has demonstrated exceptional accuracy and efficiency in automatically measuring fetal biometry, gestational age, and fetal weight. This automatic intelligent measurement software provides a rapid and precise method for evaluating fetal growth and development.

## Data Availability

The datasets and code are not publicly available due to the hospital policy and personal privacy but are available from the corresponding author on reasonable request.

## Abbreviations

AC: Abdominal circumference
AI: Artificial intelligence
BA: Bland-Altman
BMI: Body mass index
BPD: Biparietal diameter
CNS: Central nervous system
DVP: Deepest vertical pocket of amniotic fluid
EFW: Estimated fetal weight
FGR: Fetal growth restriction
FL: Femoral length
GA: Gestational age
HC: Head circumference
HL: Humeral length
ICC: Intra-class correlation coefficient
ISUOG: International Society of Ultrasound in Obstetrics and Gynecology
LGA: Large for gestational age
LVW: Lateral ventricles width
MAE: Mean absolute error
OFD: Occipitofrontal diameter
PCC: Pearson correlation coefficient
PCFW: Posterior cranial fossa pool width
PE: Percentage error
SGA: Small for gestational age
TCD: Transverse cerebellar diameter

## Footnotes

1 https://github.com/nvidia-holoscan/holoscan-docs/blob/master/devkits/clara-agx/clara_agx_user_guide.md
